# Light‐activatable minimally invasive ethyl cellulose ethanol ablation: Biodistribution and potential applications

**DOI:** 10.1002/btm2.10696

**Published:** 2024-07-12

**Authors:** Jeffrey Yang, Chen‐Hua Ma, John A. Quinlan, Kathryn McNaughton, Taya Lee, Peter Shin, Tessa Hauser, Michele L. Kaluzienski, Shruti Vig, Tri T. Quang, Matthew F. Starost, Huang‐Chiao Huang, Jenna L. Mueller

**Affiliations:** ^1^ Fischell Department of Bioengineering University of Maryland College Park Maryland USA; ^2^ Center for Interventional Oncology, Radiology and Imaging Sciences NIH Clinical Center, National Cancer Institute, National Institutes of Health Bethesda Maryland USA; ^3^ Laboratory of Cell Biology Center for Cancer Research, National Cancer Institute, National Institutes of Health Bethesda Maryland USA; ^4^ Division of Veterinary Resources Office of Research Services, National Institutes of Health Bethesda Maryland USA; ^5^ Stewart Greenebaum Cancer Center, University of Maryland School of Medicine Baltimore Maryland USA

**Keywords:** ethanol ablation, ethyl cellulose, fluorescence imaging, intratumoral injection, photodynamic therapy, photosensitizer

## Abstract

While surgical resection is a mainstay of cancer treatment, many tumors are unresectable due to stage, location, or comorbidities. Ablative therapies, which cause local destruction of tumors, are effective alternatives to surgical excision in several settings. Ethanol ablation is one such ablative treatment modality in which ethanol is directly injected into tumor nodules. Ethanol, however, tends to leak out of the tumor and into adjacent tissue structures, and its biodistribution is difficult to monitor in vivo. To address these challenges, this study presents a cutting‐edge technology known as Light‐Activatable Sustained‐Exposure Ethanol Injection Technology (LASEIT). LASEIT comprises a three‐part formulation: (1) ethanol, (2) benzoporphyrin derivative, which enables fluorescence‐based tracking of drug distribution and the potential application of photodynamic therapy, and (3) ethyl cellulose, which forms a gel upon injection into tissue to facilitate drug retention. In vitro drug release studies showed that ethyl cellulose slowed the rate of release in LASEIT by 7×. Injections in liver tissues demonstrated a 6× improvement in volume distribution when using LASEIT compared to controls. In vivo experiments in a mouse pancreatic cancer xenograft model showed LASEIT exhibited significantly stronger average radiant efficiency than controls and persisted in tumors for up to 7 days compared to controls, which only persisted for less than 24 h. In summary, this study introduced LASEIT as a novel technology that enabled real‐time fluorescence monitoring of drug distribution both ex vivo and in vivo. Further research exploring the efficacy of LASEIT is strongly warranted.

**Translational Impact Statement**

As many solid tumors are unresectable depending on stage and location, ablative therapies serve as effective alternatives for cancer patients. Ethanol ablation is an established clinical treatment strategy for solid tumors but suffers from drug leakage and poor visualization of its biodistribution. Thus, this work successfully combines polymer‐assisted ethanol ablation with a photosensitizer to enhance drug retention and enable fluorescence‐based biodistribution tracking in vivo. Our study sets the foundation for future work in effectively treating deep‐seated tumors and translation to cancer patients in all settings.

## 1 INTRODUCTION

Cancer is quickly becoming the leading cause of death globally, with an estimated 19.3 million new cancer cases and 9.9 million cancer deaths in 2020. This burden is expected to increase to 28.4 million new cancer cases annually by 2040.^1^ While surgical resection is a mainstay of cancer treatment, many tumors are unresectable due to stage, size, location, or comorbidities. Ablative therapies, which cause local destruction of tumors, are an effective alternative to surgical excision in several settings. Furthermore, ablative technologies are less invasive, less resource‐intensive, and carry less risk for periprocedural infection compared to surgery due to their minimally invasive nature.^2^

Ethanol ablation is one such ablative treatment modality in which ethanol is directly injected into tumor nodules to induce necrosis via cytoplasmic dehydration and vascular occlusion.^3^, ^4^ It has been widely used in the clinic to treat hepatocellular carcinoma and other encapsulated tumors due to its ablative efficacy, simple procedural workflow, and cost‐effectiveness. A drawback of ethanol ablation, however, is the tendency of ethanol to leak out of the tumor and into adjacent tissue structures, particularly when applied to nonencapsulated tumors.^5^ Consequently, multiple ethanol injections and procedures are often employed to achieve complete ablation of the tumor, which increases the risk of complications.

To address the limitations associated with ethanol leakage, we previously added ethyl cellulose (EC) to ethanol injections, which forms a gel upon contact with the aqueous tissue environment. EC is a hydrophobic, biocompatible polysaccharide often incorporated in the food and pharmaceutical industries.^6^, ^7^, ^8^, ^9^ In the context of clinical research, EC‐ethanol had previously been used as a sclerosing agent for treating venous malformations, with no EC‐related adverse effects observed.^10^, ^11^ We have shown that gel formation promotes ethanol retention within the tumor and subsequently maximizes ablative efficacy in oral,^5^ liver,^12^ and breast^13^, ^14^ pre‐clinical tumor models.

While EC reduced ethanol leakage, it remains difficult to monitor the spatial distribution of EC‐ethanol in vivo, which is essential for optimizing its delivery throughout the tumor while simultaneously minimizing damage to surrounding normal tissues. Adding a fluorescent dye or contrast agent to the EC‐ethanol formulation could enable precise fluorescence visualization of the gel location. Fluorescent dyes, such as indocyanine green (ICG), 5‐aminolevulinic acid (5‐ALA), and benzoporphyrin derivative (BPD), are routinely used to identify tumor locations and extent,^15^, ^16^ including tumors of hepatic,^17^ pancreatic,^18^ and colorectal^19^ origin. One class of fluorescent probes, known as photosensitizers, has an additional ability to induce reactive oxygen species when excited with near‐infrared light, which induces cell death.^20^ All three agents can be used as fluorescent tracers and photosensitizers for imaging and photodynamic therapy applications, though 5‐ALA is a prodrug that requires intracellular heme synthesis to produce the protoporphyrin IX (PpIX) photosensitizer for imaging and therapy.^21^ One distinct advantage of BPD over ICG and 5‐ALA lies in its higher singlet oxygen quantum yield (BPD: 0.76^22^; PpIX: 0.56^22^; ICG: 0.008^23^), making BPD a superior photodynamic therapy agent.

This study presents a cutting‐edge technology known as Light‐Activatable, Sustained‐exposure Ethanol Injection Technology (LASEIT), a tripartite formulation that consists of: (1) ethanol, (2) BPD, a clinically relevant photosensitizer that enables fluorescence‐based visualization of drug distribution within tissues, and (3) ethyl cellulose, a cellulose derivative that forms a gel upon injection into aqueous media, such as tissue. The studies introduced here focused on investigating the physical properties of LASEIT, along with tracking and optimizing its biodistribution within tissue‐mimicking phantoms, ex vivo tissues, and an in vivo pancreatic tumor mouse model, each of which are described in the Materials and Methods section.

## 2 MATERIALS AND METHODS

### 2.1 Preparation of LASEIT (BPD‐EC‐ethanol)

A mixture of EC (Sigma Aldrich, St. Louis, MO), benzoporphyrin derivative (BPD, U.S. Pharmacopeia, Rockville, MD), and ethanol (200 proof, Koptec, King of Prussia, PA) was prepared by stirring at room temperature, as previously described.^5^ The ratio of EC to ethanol was 6% (w/w), while the BPD concentrations ranged between 0 and 100 μM (BPD to EC‐ethanol solution, v/v), as per previous work.^24^, ^25^ The stock concentration of BPD was determined using UV–Vis spectroscopy (Synergy Neo2, BioTek, Winooski, VT) with excitation/emission (Ex/Em) wavelengths of 435/685 nm. For photochemical characterization, Singlet Oxygen Sensor Green (SOSG; Invitrogen, Carlsbad, CA) was also added to LASEIT at a fixed concentration of 7 μM to measure fluorescence induced by BPD activation (Ex/Em: 505/525 nm), as previously described.^26^ All solution preparation and subsequent experiments were performed with minimal exposure to ambient light to minimize photoactivation of the agents throughout data acquisition.

### 2.2 Preparation of tumor‐mimicking mechanical phantoms

Agarose‐based mechanical phantoms were used to evaluate the distribution of infusion volume in vitro, as previously described.^12^ 1% agarose (UltraPure Agarose, Invitrogen, Carlsbad, CA) was dissolved in deionized water (agarose: water, w:v) and stirred over a hot plate until a clear solution was obtained. The molten agarose solution was then distributed into 20‐dram cylindrical polystyrene containers (Fisher Scientific, Hampton, NH) and allowed to solidify at 4°C overnight.

### 2.3 Injection of LASEIT into mechanical phantoms

**FIGURE 1:**
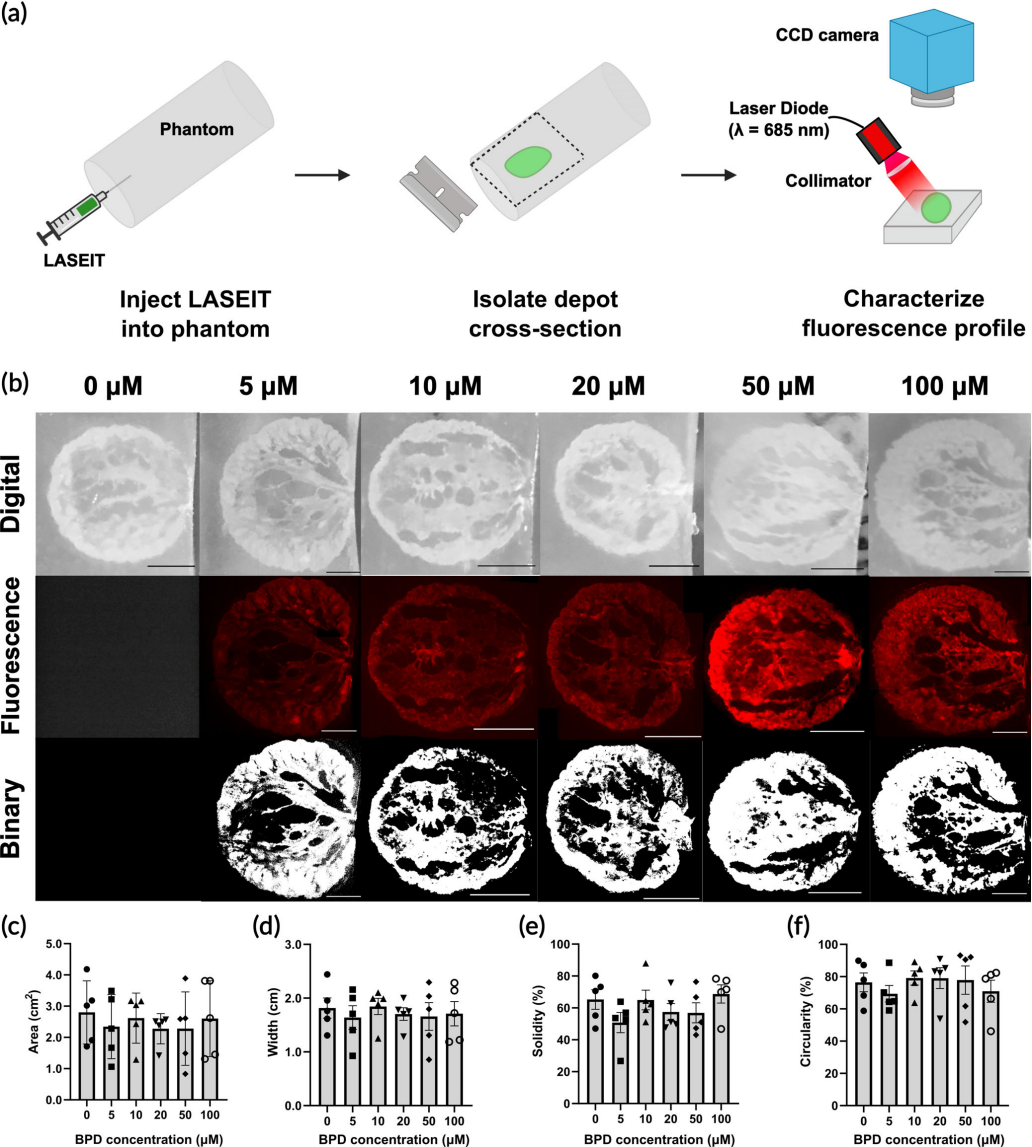
LASEIT depot formation in phantoms. (a) Workflow schematic of LASEIT injections into tissue‐mimicking phantom substrates. (b) Panel of gray‐scale digital, fluorescence, and binary images of LASEIT injections in phantoms when excited with a 685 nm laser, as a function of BPD concentration. Scale bars = 5 mm. The distribution zone of LASEIT in the phantoms is consistent across the BPD range, as evidenced by the (c) area, (d) width, (e) solidity, and (f) circularity (*n* = 5; error bars are SEM).

Figure 1a illustrates the experimental setup for performing injections into the phantoms. 3 mL syringes affixed with 27‐gauge 13‐mm length hypodermic needles were used to inject LASEIT. The syringe was connected to a programmable syringe pump (NE‐1000, New Era Pump Systems Inc., Farmingdale, NY) to control the infusion parameters (i.e., volume, rate). The needle was fully inserted into the flat surface of the phantom before starting the injection.

The resulting depot in the phantoms was sectioned, and images of the front cross‐sections of the LASEIT depot were then acquired. A 685 nm laser diode (HL6750MG, ThorLabs, Newton, NJ) delivered at 5 mW/cm^2^ was used to excite the BPD. The resulting emission was collected through a 735/728 nm bandpass filter (FF01‐735/28‐25, Semrock, West Henrietta, NY) and captured with a CCD camera situated above the specimen. An exposure time of 20 ms was used to obtain the images from each sample, unless otherwise indicated. Red pseudocoloring was applied to the fluorescence images obtained. ImageJ software (Fiji, National Institutes of Health, Bethesda, MD) was used to segment LASEIT distributions. Specifically, binary images were generated in ImageJ by applying an automated Otsu^27^ thresholding algorithm to the raw images, and the distribution areas were subsequently measured.

### 2.4 Photochemical characterization of LASEIT under low‐power fluorescence imaging conditions

**FIGURE 2:**
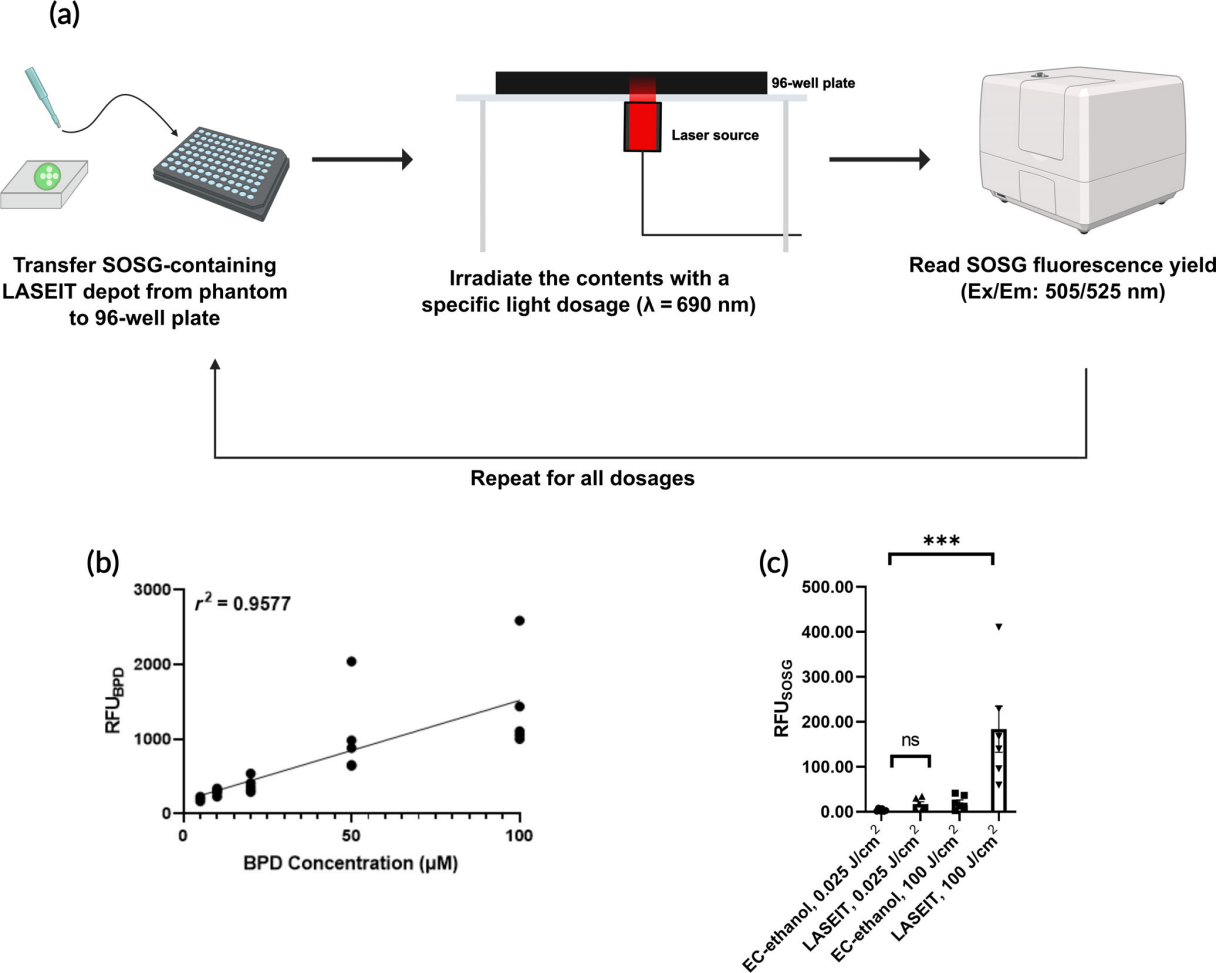
Photochemical properties of LASEIT. (a) Workflow schematic for evaluating the photochemical properties of LASEIT injected into phantoms. (b) BPD fluorescence intensity in LASEIT showed good linearity across measured BPD concentrations in the depots (*n* = 5; error bars are SEM). (c) SOSG fluorescence intensity in LASEIT remained low upon exposure to low imaging irradiation doses (*n* = 6; error bars are SEM; ns, not significant, ***P < 0.001).

Figure 2a illustrates the experimental setup for evaluating the photochemical properties of LASEIT. LASEIT containing SOSG was injected into agarose phantoms, as described earlier. Solidified LASEIT depots were removed from the phantoms, placed in a 96‐well plate, and irradiated with a 690 nm laser source at a dose equivalent to that delivered during imaging (0.025 J/cm^2^ at 5 mW) or at a therapeutically relevant dose (100 J/cm^2^ at 50 mW/cm^2^), as previously described.^24^ Light controls that did not contain BPD were also prepared in the same plate and exposed to the same irradiation dosages as their corresponding experimental groups. Additionally, dark controls were prepared—they were covered in aluminum foil for the duration of the procedure (i.e., no light exposure). A microplate reader (Synergy Neo2, BioTek) was used to acquire SOSG fluorescence signals (Ex/Em: 505/525 nm) in the specimens before and after irradiation. The fluorescence signals collected were normalized to the respective dark controls.

### 2.5 Diffusion of chemical agents from LASEIT depot

Figure 4a illustrates the experimental setup for evaluating the amount of BPD and ethanol released from LASEIT in solution. A total of 100 μL of LASEIT (at 20 μM BPD) was added to 20 mL of 10% FBS‐PBS (v/v) in a 50 mL conical tube and vortexed for 10 s at the lowest settings (Touch Mixer Model 232, Fisher Scientific, Hampton, NH). After placing the tube in a 37°C incubator, 500 μL of solution was then removed at various time points for up to 7 days, with 500 μL of fresh FBS‐PBS replenished after each collection. To measure ethanol concentration, the Amplite® Ethanol Quantitation Kit (AAT Bioquest, Pleasanton, CA) was used. Briefly, equal amounts of sample and working solution were combined and incubated at room temperature for 20 min. Fluorescence intensity was then read at Ex/Em: 540/590 nm and converted to ethanol concentration via standard curves from the same analysis and ethanol volume. The percent ethanol release was subsequently calculated by dividing the volume released at each time point by the initial volume of ethanol present in LASEIT. To measure BPD fluorescence intensity, 100 μL of each sample was placed in an all‐black, 96‐well microplate and read at Ex/Em: 435/700 nm in triplicate. BPD concentration was determined from standard curves, and then converted to nanomoles. The percent BPD release was subsequently calculated by dividing the moles released at each time point by the total moles of BPD present in LASEIT.

Figure 4c illustrates the experimental setup for examining the impact of EC on the diffusion rate of BPD. A custom‐made Franz cell (4G‐01‐01‐11.28‐08, PermeGear Inc., Hellertown, PA) was placed on top of a stirring hot plate with a stir bar added into the receptor chamber. 8 mL of ethanol solvent was added into the receptor chamber, with a 0.45 μm polyvinylidene fluoride (PVDF) membrane placed at the joint of the cell. 2 mL of LASEIT (6% EC‐ethanol, 100 μM BPD) was added into the donor compound, and the joint was secured with a cell clamp. 500 μL samples were removed from the receptor chamber at each time point and placed into 2 mL microtubes. The receptor chamber was immediately replenished with 500 μL fresh ethanol after each sampling. The solutions were distributed in triplicate into a 96‐well plate, and the fluorescence intensity was measured with a microplate reader (Synergy Neo2, BioTek, Winooski, VT) at Ex/Em: 435/685 nm (Figure S3). A standard curve of BPD concentrations ranging from 0 to 100 μM was also prepared in the same 96‐well plate to interpolate the sample fluorescence intensities measured into BPD concentration.

To calculate the diffusion coefficient (*D*) of LASEIT in the Franz cell, the following time‐lag formula^28^ was implemented: $D=\frac{h^{2}}{6t_{\mathrm{lag}}}$, where *h* is the thickness of the PVDF membrane, while *t*_lag_ is the time lag, which was the time required to reach the reservoir chamber.

### 2.6 Injection of LASEIT into excised swine liver

Figure 5a illustrates the experimental setup for performing injections of LASEIT into swine liver. For tissue experiments, fresh swine livers were excised from Yorkshire pigs (protocol approved by the University of Maryland, College Park Institutional Animal Care and Use Committee (IACUC, Protocol # R‐AUG‐20‐47). Livers were stored in Krebs‐Ringer bicarbonate buffer until ready for use (K4002, Sigma). After injection, fluorescence images of the front and side cross‐sections of the LASEIT depot were acquired and processed, as similarly described in the procedure for image acquisition in the phantoms. An exposure time of 15 ms was used to obtain the intensity readings of each sample, unless otherwise indicated. Binary images of the fluorescence images were generated in ImageJ by implementing the automatic Otsu^27^ thresholding algorithm as described above.

### 2.7 Induction of MIA PaCa‐2 tumors for LASEIT treatment

Figure 6a illustrates the experimental setup for performing injections of LASEIT into live, tumor‐bearing mice along with the accompanying analyses of the resulting depot. All animal studies were approved by the University of Maryland, College Park IACUC and were performed following ARRIVE guidelines (Protocol # R‐MAR‐22‐16). All procedures were performed under isoflurane anesthesia. Swiss nude male mice (J:Nu strain #007850, The Jackson Laboratory, Bar Harbor, ME) between 5 and 6 weeks old were used. Because these experiments focused on visualizing the biodistribution of LASEIT within the tumor microenvironment, gender is not expected to influence the interpretation of the results. MIA PaCa‐2 cells (5.0 × 10^6^ cells/animal) encased in Matrigel were subcutaneously injected into the right flank of the mice. The tumor volumetric size and mouse body weight were monitored every 2–3 days throughout the entirety of the study. Once tumors reached approximately 250 mm^3^, 60 μL of LASEIT (6% EC‐ethanol, 20 μM BPD) or controls were directly injected into the tumor. 1.5 h later, the tumors containing BPD were then irradiated (690 nm, 60 J/cm^2^, 100 mW/cm^2^). After 1 and 2 weeks post‐treatment, the mice were euthanized, and the tumors were collected for post‐mortem analysis.

### 2.8 In vivo biodistribution of LASEIT in MIA PaCa‐2 tumors post‐treatment

To evaluate the in vivo fluorescent imageability of LASEIT, mice were treated with LASEIT and observed at 0 (immediately after injection), 90 min, and 24 h post‐treatment using an IVIS Spectrum in vivo imaging system (PerkinElmer, Waltham, MA) (Ex/Em: 430/700 nm, 500 ms exposure time, 8 binning, f/2 aperture, 6.6 field of view). All mice were euthanized after acquiring the images pertaining to the 24‐h time point. All images contained standard concentrations of BPD, which were used to help scale the fluorescence signal across treatment groups. The regions of interest (ROI), depicting the depot contours, were automatically detected in the Living Image software (PerkinElmer) based on locating peak pixel intensities and searching the neighborhood around a peak pixel. The average radiant efficiency and distribution area of the depots for each tumor was subsequently measured in the Living Image® software.

### 2.9 Histopathology assessment of MIA PaCa‐2 tumors treated with LASEIT

Harvested tumors were snap frozen and sectioned into 10‐μm slices with a cryostat (CM1950, Leica Microsystems, Buffalo Grove, IL). Alternating slices were then stained with hematoxylin & eosin (H&E) and visualized under brightfield microscopy (DMi8, Leica Microsystems) at a magnification of 10×. Unstained slides were also imaged with a fluorescence microscope (Lionheart FX, BioTek) (Ex/Em: 445/685 nm) at 10× magnification to visualize any BPD fluorescence retained within the vicinity of the ablation zone.

### 2.10 Statistical analysis

Unless specified otherwise, for LASEIT physical characterization analysis in phantoms, the Kruskal‐Wallis non‐parametric analysis of variance was performed to compare any significant differences between three or more groups. The Dunn's non‐parametric multiple comparisons test was subsequently used when differences were detected. For comparing any significant differences between two groups (i.e., LASEIT and BPD‐ethanol control group in phantoms and liver, LASEIT and BPD‐PBS control group in the IVIS experiments), the Mann–Whitney U test was performed. A significance level of *p* = 0.05 was applied to reject the null hypothesis in all analyses.

## 3 RESULTS

### 3.1 LASEIT depots are imageable under fluorescence microscopy and show BPD is retained within the EC depot

Figure 1 illustrates the spatial distribution of LASEIT when injected into agarose phantoms at an infusion rate of 30 mL/h and infusion volume of 300 μL. The EC‐ethanol concentration was set to 6% (w/v), while the BPD content varied between 0 and 100 μM. As LASEIT traveled through the phantom during the infusion process, the EC solidified and settled into a rigid depot with clear boundaries along the gel front. Representative digital, fluorescence, and binary images of the depot demonstrate its visibility upon both visual inspection and under BPD excitation, respectively (Figure 1b). The coverage area and widths of the depots across all BPD concentrations averaged 2.5 ± 0.2 cm^2^ and 1.7 ± 0.1 cm (mean ± S.E.M., *n* = 30; Figure 1c,d), respectively, while their solidity and circularity spanned between 61% ± 3% (Figure 1e) and 75% ± 2% (Figure 1f), respectively. The uniformity of the depots' distribution characteristics across BPD concentrations suggests that BPD is well localized within the EC depot after injection in the phantoms.

### 3.2 LASEIT depots do not generate significant levels of singlet oxygen until specifically activated

Figure 2 shows the photochemical properties of LASEIT injected into phantoms. A best fit line was applied to the fluorescence intensity signals obtained from various concentrations of BPD (5–100 μM of BPD dissolved in 6% EC‐ethanol); the coefficient of determination was 0.9577, which suggests good linearity across the BPD concentrations (Figure 2b). Due to this observed trend and consistent distribution areas, all subsequent experiments, unless otherwise specified, implemented a BPD concentration of 20 μM. The fluorescence intensity of SOSG in LASEIT after exposure to 0.025 J/cm^2^ of NIR light (i.e., an imaging dose) was 3.98 ± 0.86 RFU (mean ± S.E.M., *n* = 5), which was not significantly different from controls (Figure 2c). Conversely, the fluorescence intensity of SOSG in LASEIT after exposure to 100 J/cm^2^ (i.e., a therapeutic dose) was 50.12 ± 30.79 RFU, which was significantly higher than the imaging dose and controls, suggesting that imaging LASEIT depots does not generate significant levels of SOSG.

### 3.3 Injection parameters influence LASEIT distribution

**FIGURE 3:**
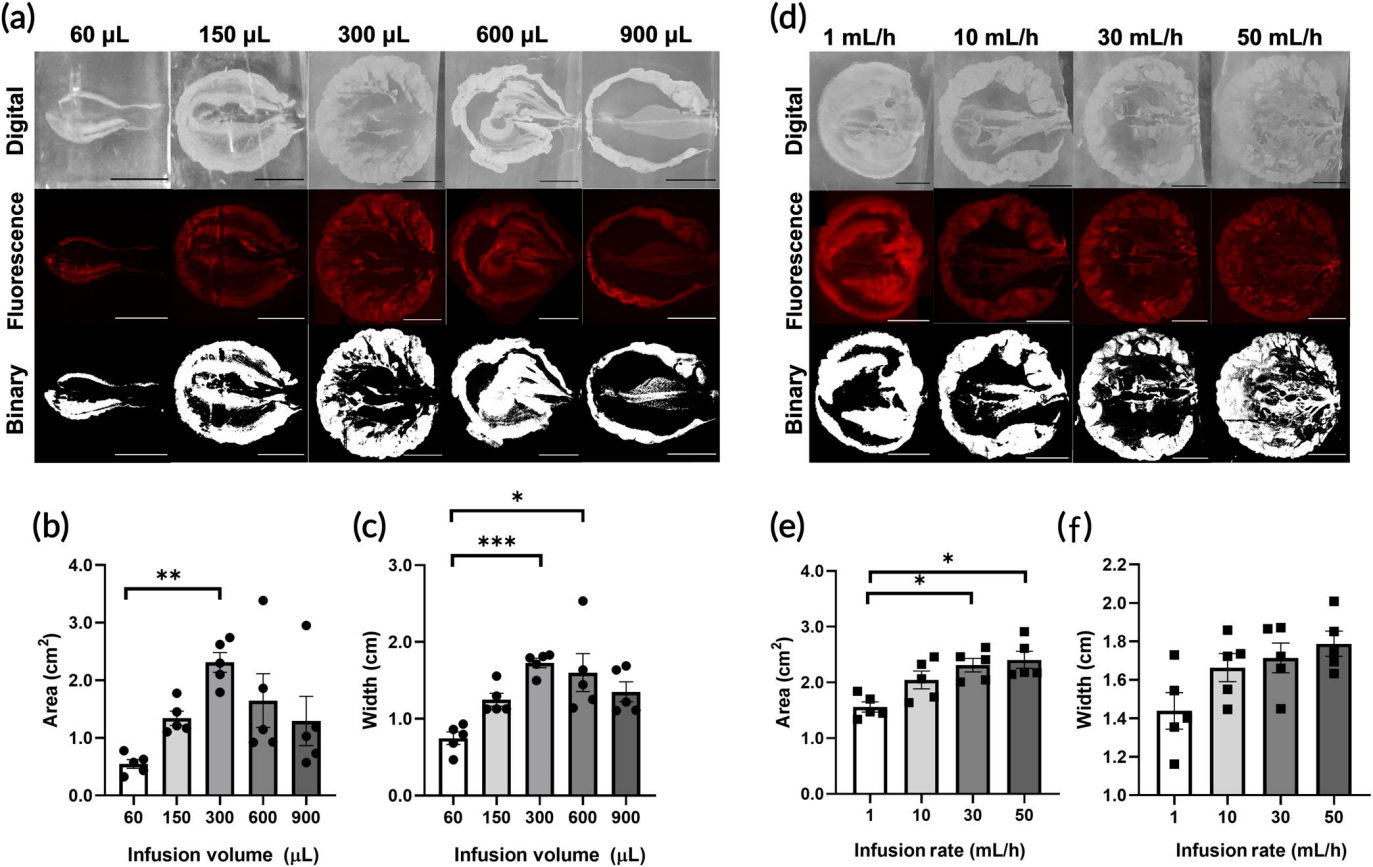
LASEIT distribution area as a function of infusion parameters. (a) Panel of representative fluorescence and digital images of LASEIT injections (20 μM) as a function of infusion volume. The infusion rate was kept constant at 30 mL/h. (b) LASEIT distribution area and (c) depot width as a function of infusion volume (*n* = 5). (d) Panel of representative fluorescence and digital images of LASEIT injections as a function of infusion rate. The infusion volume was kept constant at 300 μL. (e) LASEIT distribution area and (f) depot width as a function of infusion rate (*n* = 5). All error bars in this figure are SEM. *P < 0.05; **P < 0.01; ***P < 0.001.

Figure 3 illustrates the distribution of LASEIT in phantoms as a function of various infusion volumes (60–900 μL) and infusion rates (1–50 mL/h), as per previous work.^12^, ^29^ Representative digital, fluorescence, and binary images of the depots varying infusion volumes while keeping rate constant at 30 mL/h (Figure 3a) and depots varying infusion rates while keeping volume constant at 300 μL (Figure 3d) are shown. Distribution area increased with infusion volume up to 300 μL (Figure 3b), which achieved the largest average distribution area of 2.3 ± 0.2 cm^2^ (mean ± S.E.M., *n* = 5). Beyond this point, the 600 and 900 μL showed a decrease in distribution area. Similar trends were observed for the width of the depot (Figure 3c), with the 300 μL group achieving the largest depot width of 1.7 ± 0.1 cm (mean ± S.E.M., *n* = 5). Distribution area also increased with infusion rate (Figure 3e), though the distribution area started to plateau at higher rates, achieving an area of 2.3 ± 0.1 and 2.4 ± 0.2 cm^2^ (mean ± S.E.M., *n* = 5) for 30 and 50 mL/h, respectively. Similar trends were observed for the width of the depot (Figure 3f), though no significant differences were observed. These observations suggest that an infusion volume of 300 μL and infusion rates of 30 mL/h and above achieve maximal distribution area in phantoms.

### 3.4 LASEIT retains chemical agents and delays short‐term drug release

**FIGURE 4:**
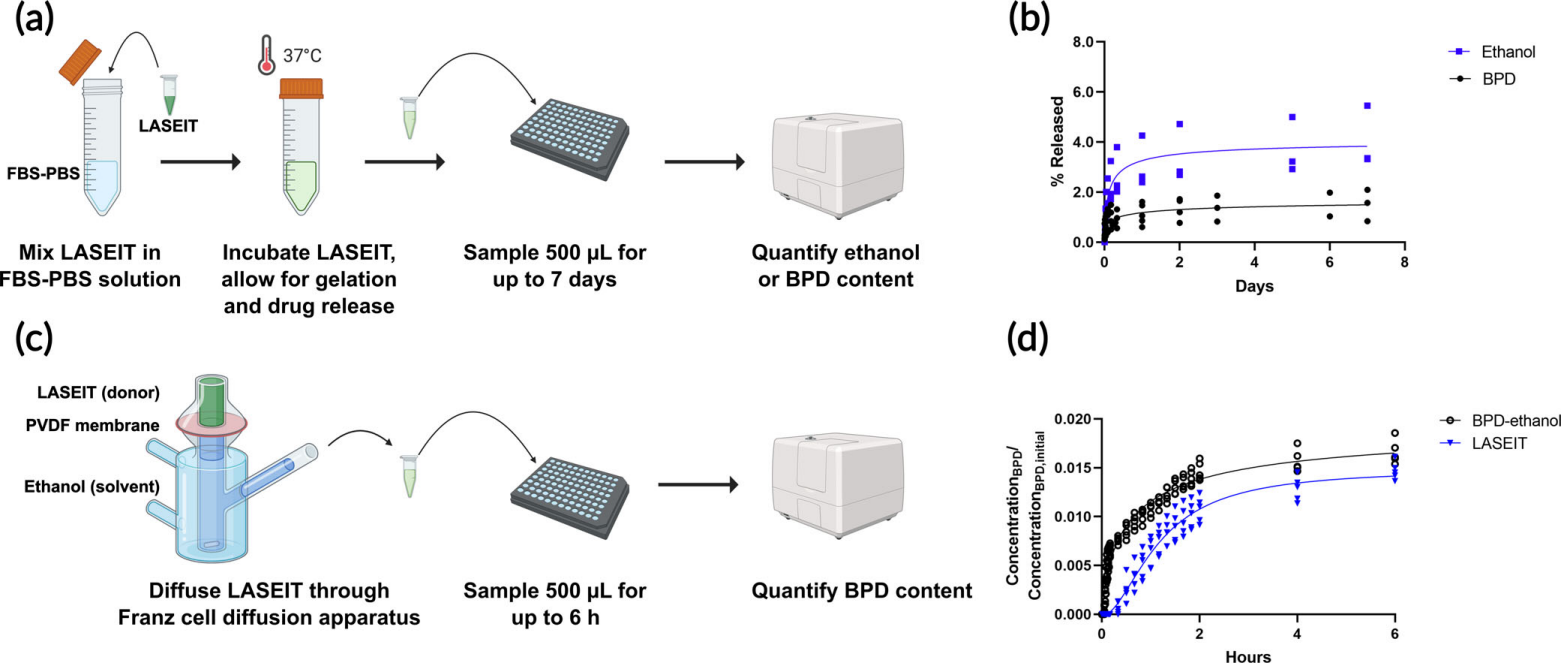
Release kinetics of LASEIT in vitro. (a) Workflow schematic for evaluating drug release from LASEIT in aqueous solution under static conditions. (b) Percent release of BPD and ethanol from LASEIT statically submerged in FBS‐PBS over time. (c) Workflow schematic for evaluating drug release from LASEIT in ethanol under sink conditions. (d) Ratio of concentration of BPD released to initial BPD concentration within the Franz cell over time. The diffusion coefficients for BPD‐ethanol and LASEIT were calculated to be 1.9 × 10^−10^ and 2.9 × 10^−11^ cm^2^/s, respectively.

Figure 4 shows the in vitro release kinetics of LASEIT in both static and sink conditions. For the experiment under static conditions, LASEIT was exposed to FBS‐PBS over a 7‐day period. There was an immediate increase in BPD and ethanol release from the depot within the first 8 h (up to 2.7% and 0.9% total release, respectively), before quickly plateauing and ending at 4.0% and 1.5% total release, respectively, at day 7 (Figure 4b). All remaining BPD and ethanol were retained within the LASEIT depot. For the experiment under sink conditions, LASEIT or controls (BPD‐ethanol) was placed in the donor compartment of a Franz cell and its diffusion through a membrane was sampled over a 24‐h period. The presence of EC substantially slowed diffusion for LASEIT, compared to that of BPD‐ethanol (Figure 4d). Notably, the diffusion coefficient for LASEIT was 2.9 × 10^−11^ cm^2^/s, while that for BPD‐ethanol was 1.9 × 10^−10^ cm^2^/s, which corroborates the slower rate of release observed for LASEIT compared to that of BPD‐ethanol.

### 3.5 LASEIT is imageable in liver tissue and increases distribution volume sixfold compared to control

**FIGURE 5:**
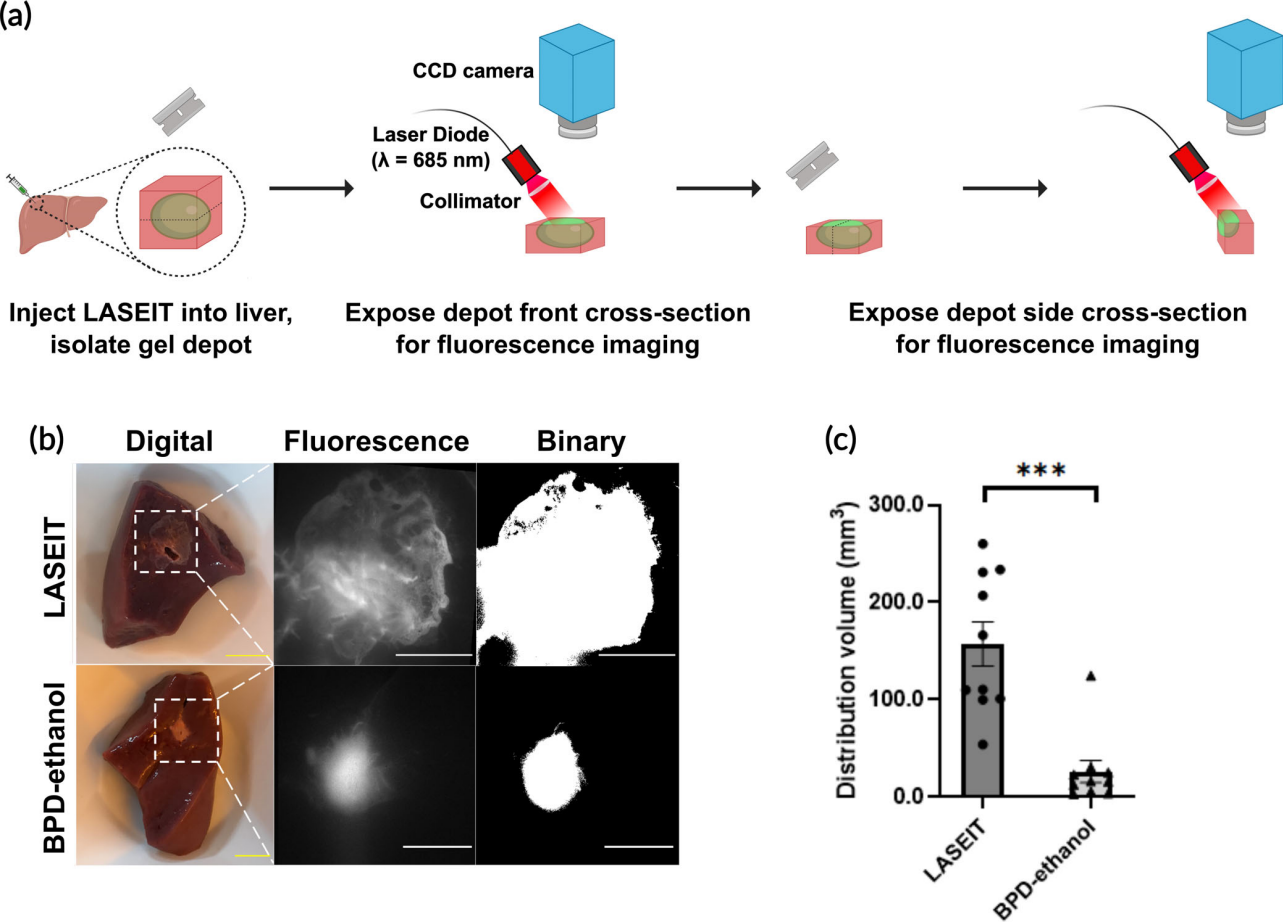
LASEIT distribution volume in liver tissue. (a) Workflow schematic of LASEIT injections into excised swine liver. (b) Representative digital, fluorescence, and segmented binary images of both LASEIT (top row) and control (bottom row) in excised swine liver. Yellow scale bars = 10 mm; white scale bars = 5 mm. (c) LASEIT significantly increased the distribution volume in the tissues, compared to the BPD‐ethanol control (*n* = 10). Error bars are SEM. ***P < 0.001.

Figure 5 illustrates the distribution of LASEIT in swine livers, notably in direct comparison to the BPD‐ethanol depot. Representative digital, fluorescence, and binary images of the front cross‐sections of the LASEIT and BPD‐ethanol depots are shown in Figure 5b. The BPD concentration used for both formulations was 20 μM, while the injection parameters chosen were 300 μL volume and 30 mL/h rate. The distribution volume achieved by the LASEIT injections was 157.3 ± 22.6 mm^3^ (mean ± S.E.M., *n* = 10), which was significantly larger than the volume achieved by the BPD‐ethanol injections (25.9 ± 11.4 mm^3^; mean ± S.E.M., *n* = 10; Figure 5c), illustrating how EC increased distribution volume sixfold in tissue compared to that of the control.

### 3.6 LASEIT fluorescence enables in vivo depot visualization within MIA PaCa‐2 tumors 24 h post‐treatment

**FIGURE 6:**
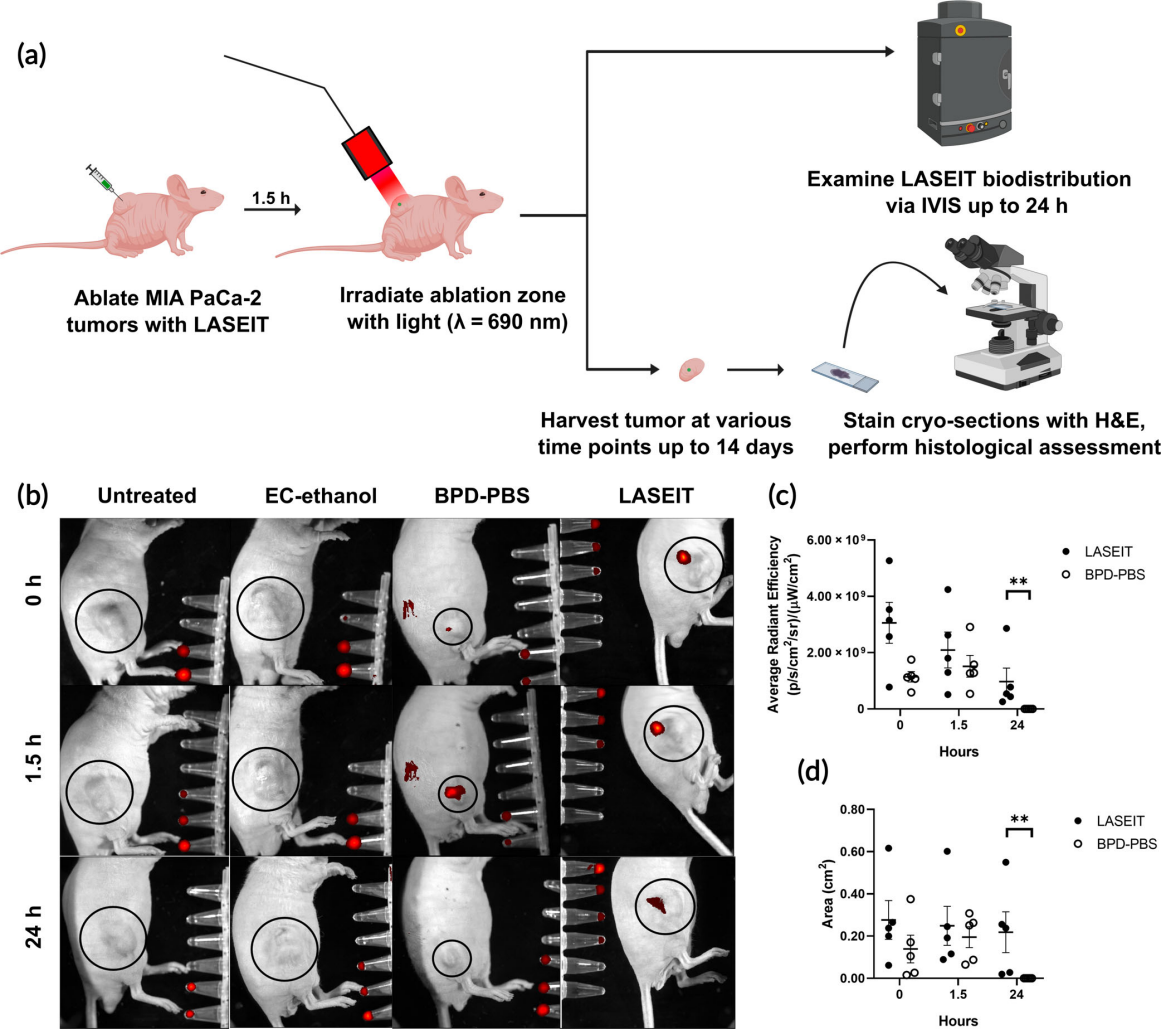
LASEIT fluorescence in MIA PaCa‐2 tumors is detectable with IVIS 24 h post‐treatment. (a) Workflow schematic for LASEIT injections in tumor‐bearing mice and either evaluating LASEIT biodistribution or conducting histopathological analysis. (b) Representative IVIS images of LASEIT injections and associated controls over a span of 24 h (Ex/Em: 430/700 nm). (c) Average radiant efficiencies of LASEIT and BPD‐PBS over a span of 24 h. (d) Distribution area of LASEIT and BPD‐PBS depots over a span of 24 h (*n* = 5). Error bars are SEM. **P < 0.01.

Figure 6 displays the depot fluorescence profile of LASEIT in the MIA PaCa‐2 tumors over 24 h. Representative IVIS images of all treatment groups for the 0‐, 1.5‐, and 24‐h time points are shown in Figure 6b. The LASEIT and BPD‐PBS groups showed a strong fluorescence signal within the tumor site at the 0‐ and 1.5‐h time points, though only the LASEIT group exhibited any detectable fluorescence signal at the 24‐h time point. No fluorescence signal was detected at any time point for the EC‐ethanol and untreated groups. The average radiant efficiencies and distribution areas were measured for the LASEIT and BPD‐PBS groups (Figure 6c,d, respectively). Compared to the BPD‐PBS group, LASEIT showed higher average radiant efficiency and larger distribution area at both the 0‐h time point (average radiant efficiency: 3.06 × 10^9^ vs. 1.14 × 10^9^; distribution area: 0.28 vs. 0.14 cm^2^) and 1.5‐h time point (average radiant efficiency: 2.09 × 10^9^ vs. 1.51 × 10^9^; distribution area: 0.25 cm^2^ vs. 0.19 cm^2^). No major fluctuations in the first 90 min were observed for LASEIT; conversely, an increase in both average radiant efficiency and distribution area was observed in the BPD‐PBS group after 90 min. Both groups saw a significant decrease in average radiant efficiency and distribution area by the 24‐h time point, though the LASEIT group still had some signal detected by IVIS.

### 3.7 LASEIT retains BPD in its depot over 1 week

**FIGURE 7:**
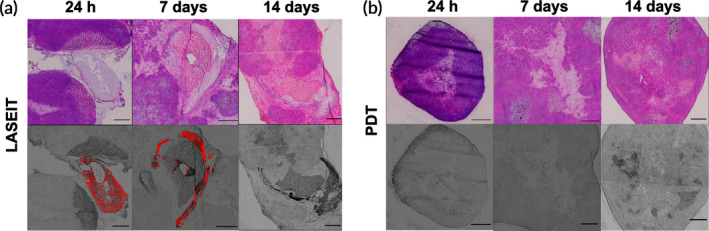
Histopathology assessment of the LASEIT ablation zone within MIA PaCa‐2 tumors. (a,b) Representative H&E and fluorescence images of tumor slides treated with LASEIT or its monotherapy counterparts. BPD was present and detectable in the LASEIT group up to 7 days post‐treatment, while BPD was undetectable by the 24‐h time point in the PDT group. Black scale bars = 1 mm.

Figure 7 displays the H&E and fluorescence images of the ablation zone induced by the BPD‐containing treatment groups, LASEIT and photodynamic therapy (PDT), within individual sections of the MIA PaCa‐2 tumors over 14 days. As seen, BPD was detectable within the ablation zone in the LASEIT treatment group for up to 7 days, before disappearing at 14 days post‐ablation (Figure 7a). Conversely, the PDT treatment group did not have any detectable BPD signal throughout the given timeframe, even at 24 h post‐ablation (Figure 7b). None of the non‐BPD‐containing treatment groups displayed any detectable BPD signal (Figure S5).

## 4 DISCUSSION

While ethanol ablation has been used clinically to treat a variety of unresectable tumors, ethanol has the tendency to leak out of the tumor and into adjacent tissue structures. Additionally, the biodistribution of ethanol, which directly impacts safety and efficacy, is difficult to monitor in vivo. To address these challenges, we developed Light‐Activatable, Sustained‐exposure Ethanol Injection Technology (LASEIT), which combines: (1) ethanol, (2) ethyl cellulose (EC), which forms a gel upon injection to increase drug retention, and (3) BPD, a fluorescent photosensitizer to enable tracking of drug distribution. We explored the feasibility and benefits of LASEIT—specifically the benefits of gelation (achieved with EC) and fluorescence‐guided therapy (achieved with BPD) through in vitro assays and experiments in tissue‐mimicking phantoms, ex vivo tissue, and an in vivo pancreatic tumor mouse model.

The EC component of LASEIT led to gel formation around the injection site, which yielded clear benefits. First, LASEIT slowed the release rate and diffusion from the depot compared to the formulations without EC. Less than 5% of the total BPD and ethanol were released from the depot after 7 days (Figure 4b), signifying that the vast majority of the drugs were retained in the depot. Additionally, studies in Franz cells showed that EC slowed diffusion by 7× (Figure 4d). This containment and retention phenomenon is further corroborated by the detection of BPD within the injection site of LASEIT over a period of 7 days, compared to less than 24 h for controls (Figure 7). Second, LASEIT formed significantly larger distributions than the formulations without EC. This is evidenced by the lack of depot formation for BPD‐ethanol and BPD‐PBS in the phantoms (Figure S1) and the 6× larger distribution volume of LASEIT in the swine liver, compared to that of BPD‐ethanol (Figure 5). This suggests that our LASEIT formulation could deliver the same amount of drug (i.e., ethanol, BPD) as controls, but achieve 6× larger tumor coverage, thereby simultaneously improving biosafety and biodistribution. This 6× increase in distribution volume could be further enhanced through the tuning of key infusion parameters, namely infusion volume and infusion rate (Figure 3), which will be explored in future in vivo studies.

The BPD component of LASEIT provided clear visualization of drug distribution, which could enable fluorescence‐guided therapy. Fluorescence‐guided therapy offers a strong visual aid that provides high contrast and spatiotemporal resolution of drug distribution within the tumor, which could enable clinicians to quickly assess if the tumor has been adequately treated during a procedure.^30^, ^31^, ^32^ For this study, we implemented BPD as our photosensitizer fluorescent agent, due to its high singlet oxygen quantum yield and its ability to image deeper depths in tissue, thanks to its absorption band at longer wavelengths. We found that adding BPD enables fluorescent visualization of the depot for its monitoring across tissue substrates. All concentrations of BPD tested in tissue‐mimicking phantoms showed detectable levels of fluorescence signal, which was localized within the EC‐ethanol depot (Figure 1). The imageability of BPD within the LASEIT depot was preserved in swine liver (Figure 5), a key advantage of LASEIT, as fluorescence imaging is often hindered by autofluorescence and absorbance of pigmented tissues.^33^, ^34^ Furthermore, BPD allowed for fluorescence monitoring of the LASEIT depot in vivo through the skin (Figure 6), which could have significant implications for real‐time assessment of superficial tumor coverage.^35^ Of note is the stronger fluorescence intensity observed for LASEIT, compared to that of the BPD‐PBS group (Figure 6c); this phenomena is likely due to the presence of ethanol, which discourages BPD aggregate formation.^36^ Additionally, we found that repeated illumination of BPD during fluorescence imaging sessions in phantoms (Figure 2c) and in vivo (Figure S4) did not lead to photobleaching^37^ or premature production of singlet oxygen. Taken together, the BPD component of LASEIT enabled high contrast fluorescence‐guided tracking of LASEIT biodistribution ex vivo and in vivo.

BPD also provides the potential for inducing cell death through its ability to generate a high yield of singlet oxygen when excited with therapeutic doses of red light.^38^, ^39^ This phenomenon, referred to as PDT, opens up avenues to investigate its combined tumor ablative efficacy with ethanol ablation in LASEIT. One of the challenges of delivering BPD into tissue lies in its hydrophobic nature, including its tendency to aggregate and self‐quench in aqueous solution.^40^, ^41^, ^42^ Ethanol appears to discourage BPD aggregate formation and consequently may extend the half‐life of singlet oxygen.^43^ The addition of EC as the gelling agent, which is also hydrophobic in nature, localizes both BPD and ethanol within the injection site. This may further prolong the interaction between ethanol and BPD, thereby increasing the ablative efficacy of LASEIT. Taken together, this suggests a potential synergistic effect between both therapies to enhance photochemical activity and promote cell kill, which will be investigated in future studies.

In addition to exploring the tumor ablative activity and therapeutic efficacy of LASEIT in vivo, there exist additional opportunities for future work. First, while the xenograft model provided key insights into the local effects of LASEIT on the tumor landscape, we will investigate the impact of LASEIT on the tumor microenvironment and immune system in orthotopic^44^ models or chemically induced^45^, ^46^ models. Another opportunity for future work is to study the impact of EC concentration on biodistribution. Our previous studies have shown that EC concentration can impact gel formation,^5^, ^12^, ^13^, ^14^, ^47^, ^48^ therefore in future studies we will vary the EC content in LASEIT to assess its impact on distribution volume, necrotic volume, and degradation. A third opportunity for future work is to conduct in vivo experiments explicitly evaluating the ethanol release kinetics from LASEIT, as this study only investigated the diffusive abilities of ethanol and BPD from the gel in an in vitro context. Blood alcohol levels of all subsequent animal studies will also be monitored, in addition to tracking any adverse events. Lastly, while IVIS can be used to adequately visualize BPD in flank tumors, future applications will likely require the ability to image deeper tumors within the body.^49^ Thus, future work will include developing LED‐based fiber optic imaging systems to deliver light and image intratumorally.

An important consideration for cancer research pertains to the translatability of technologies into low‐ and middle‐income countries (LMICs), as over 70% of global cancer‐related deaths now occur in LMICs.^1^ While radiotherapy and surgical resection of solid tumors are foundational methods for cancer management, up to 90% of patients in LMICs do not have access to surgical care.^50^, ^51^, ^52^ Compared to surgery, ablative therapies are less invasive, less resource‐intensive, and carry less risk for periprocedural infection due to their minimally invasive nature. However, many forms of ablation, such as radiofrequency or microwave ablation, remain inaccessible in LMICs due to the high procedural cost and need for specialized instrumentation to operate and sterilize equipment.^2^ We anticipate that LASEIT has the ability to be more affordable than these existing technologies, as many of the components are low‐cost, particularly ethanol and EC. Additionally, the clinical costs associated with PDT are decreasing over time, making them more amenable to LMIC environments. For example, other groups are currently exploring PDT as a low cost therapy to treat oral cancer.^53^ We anticipate the low doses of BPD needed for intratumoral administration will further decrease cost. Thus, from a global perspective, implementing LASEIT for the ablation of solid tumors may carry significant ramifications for cancer patients worldwide, particularly those situated in regions with limited access to medical resources.

## 5 CONCLUSIONS

LASEIT integrates ethanol ablation with fluorescence imaging into a combinatorial ablative therapy designed for ample drug depot visualization while locally ablating tumors. The increased size of the ablation zone and visibility under fluorescence imaging over a one‐week period highlights the markedly improved performance of LASEIT over its monotherapy counterparts. Such enhancements were due to the gelation abilities of EC, fluorescent properties of BPD, and inherent compatibility between EC, BPD, and ethanol to augment the ablative efficacy of LASEIT, all while reducing leakage to neighboring normal tissues. This study sets the foundation for future work optimizing LASEIT for treatment of deep‐seated tumors and translation to cancer patients.

## AUTHOR CONTRIBUTIONS

**Jeffrey Yang:** Conceptualization; data curation; formal analysis; investigation; methodology; project administration; software; supervision; validation; visualization; writing – original draft; writing – review and editing. **Chen‐Hua Ma:** Conceptualization; data curation; formal analysis; investigation; methodology; project administration; software; validation; visualization; writing – original draft; writing – review and editing. **John A. Quinlan:** Data curation; formal analysis; investigation; methodology; software; validation; visualization; writing – review and editing. **Kathryn McNaughton:** Data curation; formal analysis; investigation; methodology; validation; visualization; writing – review and editing. **Taya Lee:** Data curation; formal analysis; investigation; methodology; visualization; writing – review and editing. **Peter Shin:** Data curation; formal analysis; investigation; methodology; writing – review and editing. **Tessa Hauser:** Data curation; formal analysis; investigation; methodology; writing – review and editing. **Michele L. Kaluzienski:** Formal analysis; investigation; methodology; validation; writing – review and editing. **Shruti Vig:** Formal analysis; investigation; methodology; software; validation; writing – review and editing. **Tri T. Quang:** Formal analysis; investigation; methodology; software; validation; visualization; writing – review and editing. **Matthew F. Starost:** Data curation; formal analysis; investigation; methodology; resources; software; validation; visualization; writing – review and editing. **Huang‐Chiao Huang:** Conceptualization; data curation; formal analysis; funding acquisition; investigation; methodology; project administration; resources; supervision; validation; visualization; writing – original draft; writing – review and editing. **Jenna L. Mueller:** Conceptualization; data curation; formal analysis; funding acquisition; investigation; methodology; project administration; resources; supervision; validation; visualization; writing – original draft; writing – review and editing.

## FUNDING INFORMATION

This work is supported by the National Institutes of Health R00CA234455 (J.L.M.), R21EB028508, and R01CA260340 (H.H.) grants, the University of Maryland startup funds, and the NCI/UMD Partnership for Integrative Cancer Research.

## CONFLICT OF INTEREST STATEMENT

The authors have no conflicts of interest to declare.

## Supporting information


**Figure S1.** Individual chemical agents injected into agarose phantoms produced no detectable fluorescence signals. Pure ethanol, BPD dissolved in PBS, and BPD dissolved in pure ethanol do not generate any discernable fluorescence signal, due to the vast majority of the injectate leaking out of the phantom (as shown in the digital images).
**Figure S2.** Study workflow for assessing EC stability in solution. (a) Schematic of studying EC content within the LASEIT depot when exposed to various media. (b) Mass measurements of EC lost in water over time, in physiological conditions (*n* = 7). Error bars = S.E.M.
**Figure S3.** Release profiles of BPD from LASEIT, in terms of fluorescence intensity as a function of time. The raw fluorescence intensity was converted into concentration thanks to established standard curves at the time of signal acquisition.
**Figure S4.** BPD was not significantly photobleached after multiple IVIS imaging sessions. Fluorescence was measured for (a) 5, (b) 2, (c) 1, (d) 0.5, (e) 0.2, (f) 1, and (g) 0 μM BPD standard curve concentrations after 0, 1, 5, or 25 IVIS exposures (Ex/Em: 430/700 nm). Each tested BPD concentration maintained a similar level of fluorescence after up to 25 independent exposures to the IVIS readings (*n* = 3). Fluorescence was quantified after exposures via a plate reader (Ex/Em: 435/685 nm). No significant differences were detected. Error bars = S.E.M.
**Figure S5.** Only the LASEIT and PDT groups observed detectable BPD signals. None of the non‐BPD‐containing treatment groups exhibited BPD fluorescence. Red arrow denotes region containing BPD. Black scale bars = 1 mm.

## Data Availability

The data that support the findings of this study are available from the corresponding author upon reasonable request.
