# Supplementary material for: Light‐activatable minimally invasive ethyl cellulose ethanol ablation: Biodistribution and potential applications
Source: Bioeng Transl Med. 2024 Jul 12;9(6):e10696. doi: 10.1002/btm2.10696 (PMC11558191; doi:10.1002/btm2.10696)
Supplement: Supplementary file 1 — Figure S1. Individual chemical agents injected into agarose phantoms produced no detectable fluorescence signals. Pure ethanol, BPD dissolved in PBS, and BPD dissolved in pure ethanol do not generate any discernable fluorescence signal, due to the vast majority of the injectate leaking out of the phantom (as shown in the digital images). Figure S2. Study workflow for assessing EC stability in solution. (a) Schematic of studying EC content within the LASEIT depot when exposed to various media. (b) Mass measurements of EC lost in water over time, in physiological conditions (n = 7). Error bars = S.E.M. Figure S3. Release profiles of BPD from LASEIT, in terms of fluorescence intensity as a function of time. The raw fluorescence intensity was converted into concentration thanks to established standard curves at the time of signal acquisition. Figure S4. BPD was not significantly photobleached after multiple IVIS imaging sessions. Fluorescence was measured for (a) 5, (b) 2, (c) 1, (d) 0.5, (e) 0.2, (f) 1, and (g) 0 μM BPD standard curve concentrations after 0, 1, 5, or 25 IVIS exposures (Ex/Em: 430/700 nm). Each tested BPD concentration maintained a similar level of fluorescence after up to 25 independent exposures to the IVIS readings (n = 3). Fluorescence was quantified after exposures via a plate reader (Ex/Em: 435/685 nm). No significant differences were detected. Error bars = S.E.M. Figure S5. Only the LASEIT and PDT groups observed detectable BPD signals. None of the non‐BPD‐containing treatment groups exhibited BPD fluorescence. Red arrow denotes region containing BPD. Black scale bars = 1 mm. [file BTM2-9-e10696-s001.docx]

Light-Activatable Minimally Invasive Ethyl Cellulose Ethanol Ablation: Biodistribution and Potential Applications

Jeffrey Yang^1,2^‡, Chen-Hua Ma^1^‡, John A. Quinlan^1,3^, Kathryn McNaughton^1^, Taya Lee^1^, Peter Shin^1^, Tessa Hauser^1^, Michele L. Kaluzienski^1^, Shruti Vig^1^, Tri T. Quang^1^, Matthew F. Starost^4^, Huang-Chiao Huang^1,5^, Jenna L. Mueller^1,5^*

^1^Fischell Department of Bioengineering, University of Maryland, College Park, MD

^2^Center for Interventional Oncology, Radiology and Imaging Sciences, NIH Clinical Center, National Cancer Institute, National Institutes of Health, Bethesda, MD

^3^Laboratory of Cell Biology, Center for Cancer Research, National Cancer Institute, National Institutes of Health, Bethesda, MD

^4^Division of Veterinary Resources, Office of Research Services, National Institutes of Health, Bethesda, MD

^5^Stewart Greenebaum Cancer Center, University of Maryland School of Medicine, Baltimore, MD

‡Denotes authors contributed equally

*Denotes corresponding author


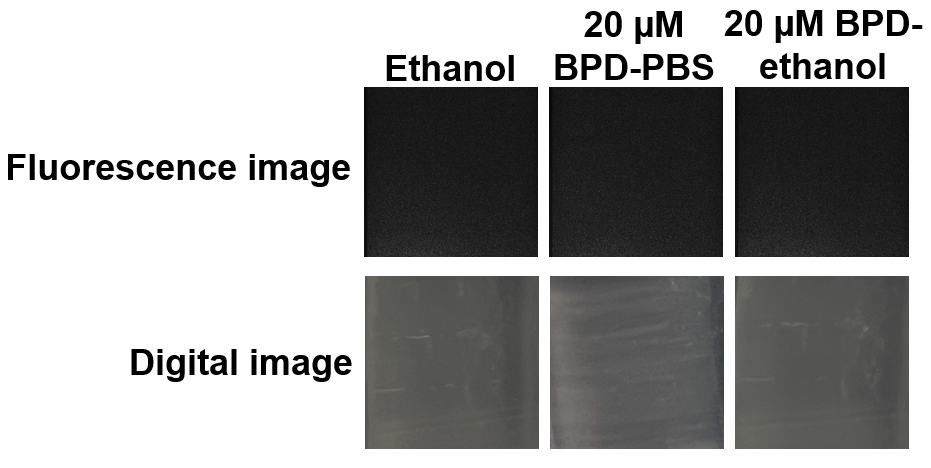


**Figure S1. Individual chemical agents injected into agarose phantoms produced no detectable fluorescence signals.** Pure ethanol, BPD dissolved in PBS, and BPD dissolved in pure ethanol do not generate any discernable fluorescence signal, due to the vast majority of the injectate leaking out of the phantom (as shown in the digital images).


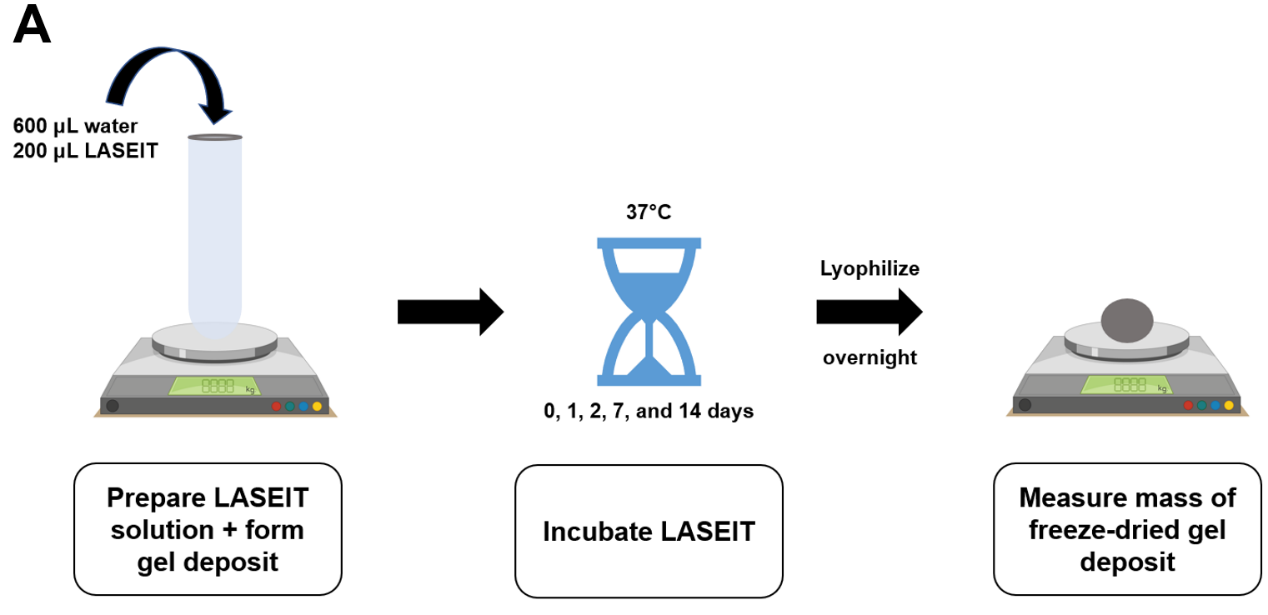


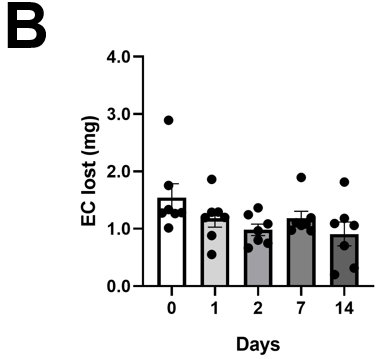


**Figure S2. Study workflow for assessing EC stability in solution.** (A) Schematic of studying EC content within the LASEIT depot when exposed to various media. (B) Mass measurements of EC lost in water over time, in physiological conditions (n=7). Error bars = S.E.M.


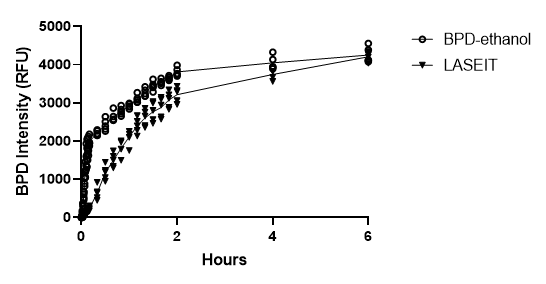


**Figure S3. Release profiles of BPD from LASEIT, in terms of fluorescence intensity as a function of time.** The raw fluorescence intensity was converted into concentration thanks to established standard curves at the time of signal acquisition (n=5).

**
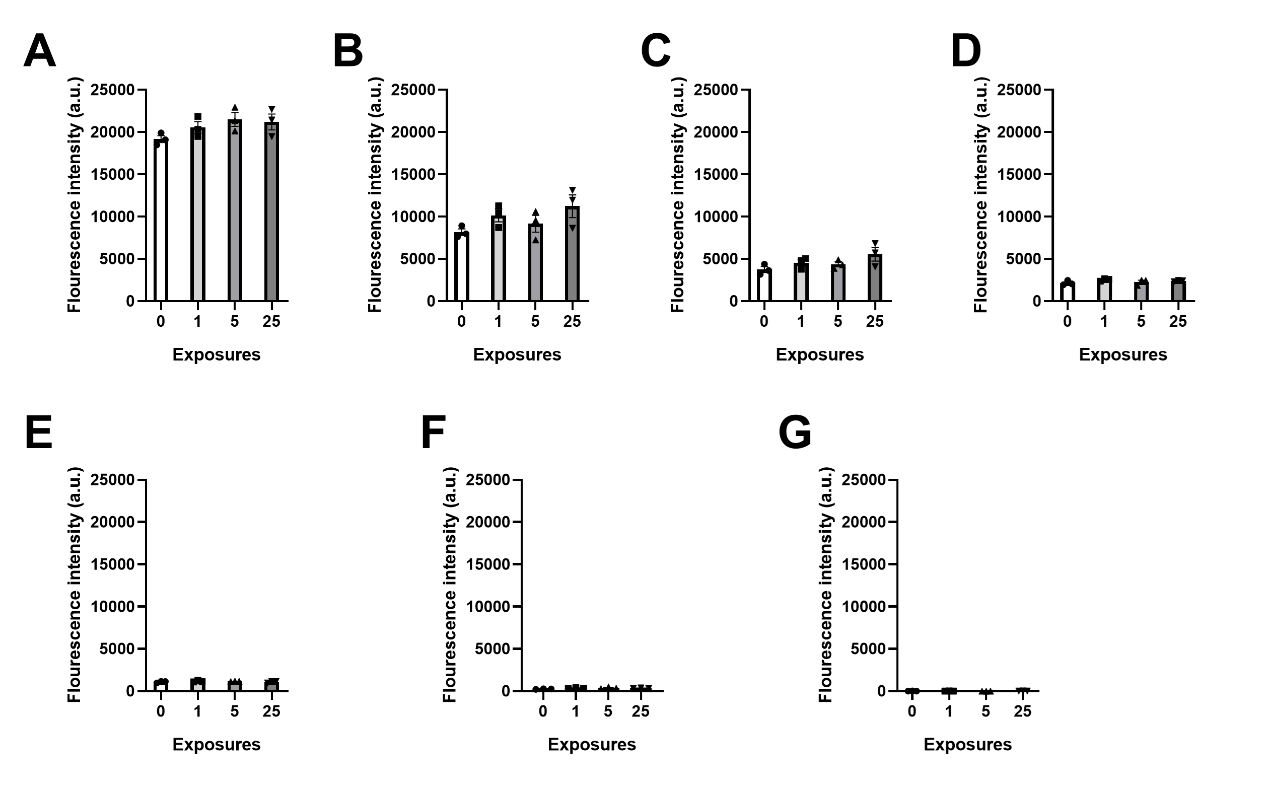
**

**Figure S4. BPD was not significantly photobleached after multiple IVIS imaging sessions.** Fluorescence was measured for (A) 5, (B) 2, (C) 1, (D) 0.5, (E) 0.2, (F) 1, and (G) 0 µM BPD standard curve concentrations after 0, 1, 5, or 25 IVIS exposures (Ex/Em: 430/700 nm). Each tested BPD concentration maintained a similar level of fluorescence after up to 25 independent exposures to the IVIS readings (n=3). Fluorescence was quantified after exposures via a plate reader (Ex/Em: 435/685 nm). No significant differences were detected. Error bars = S.E.M.


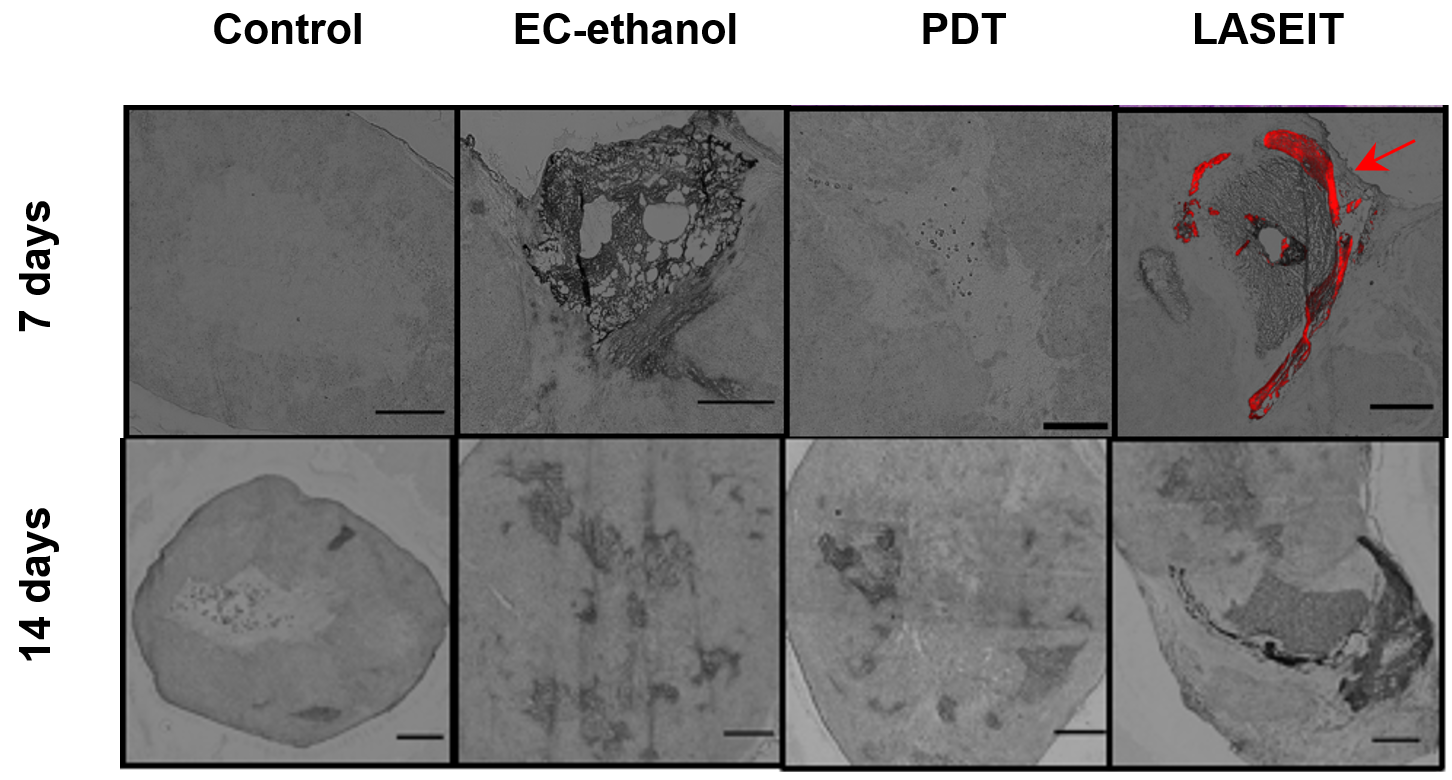
 **Figure S5. Only the LASEIT and PDT groups observed detectable BPD signals.** None of the non-BPD-containing treatment groups exhibited BPD fluorescence. Red arrow denotes region containing BPD. Black scale bars = 1 mm.
